# Are fermented foods safe for human health? A systematic analysis of the literature

**DOI:** 10.3389/fnut.2026.1905861

**Published:** 2026-08-21

**Authors:** Ricardo Assunção, Ljupco Angelovski, Zuzana Ciesarová, Pasquale Russo, Elisa Salvetti, Inga Sarand, Beatriz Nunes Silva, Aleksandra M. Torbica, Arkadiusz Zakrzewski, Müzeyyen Berkel Kasikci, Mustafa Guzel, Aline Issa, Christophe Chassard, Smilja Praćer, Guy Vergères, Marta Laranjo

**Affiliations:** 1 Food and Nutrition Department, National Institute of Health Dr. Ricardo Jorge, Lisbon, Portugal; 2 Egas Moniz Center for Interdisciplinary Research (CiiEM), Egas Moniz School of Health and Science, Almada, Portugal; 3 Faculty of Veterinary Medicine, Ss. Cyril and Methodius University, Skopje, North Macedonia; 4 National Agricultural and Food Center, Food Research Institute in Bratislava, Bratislava, Slovakia; 5 Department of Food, Environmental and Nutritional Sciences (DeFENS), University of Milan, Milan, Italy; 6 Department of Biotechnology, Verona University Culture Collection (VUCC-DBT), University of Verona, Verona, Italy; 7 Department of Chemistry and Biotechnology, Tallinn University of Technology, Tallinn, Estonia; 8 CBQF - Centro de Biotecnologia e Química Fina – Laboratório Associado, Escola Superior de Biotecnologia, Universidade Católica Portuguesa, Porto, Portugal; 9 Institute of Food Technology, University of Novi Sad, Novi Sad, Serbia; 10 Department of Food Microbiology, Meat Technology and Chemistry, University of Warmia and Mazury in Olsztyn, Olsztyn, Poland; 11 Department of Food Engineering, Faculty of Engineering and Natural Sciences, Manisa Celal Bayar University, Manisa, Türkiye; 12 Department of Food Engineering, Hitit University, Çorum, Türkiye; 13 Faculty of Nursing and Health Sciences, Notre Dame University- Louaize, Zouk Mosbeh, Lebanon; 14 Unité Mixte de Recherche sur le Fromage (UMRF 0545), Institut National de Recherche pour l'Agriculture, l'Alimentation et l'Environnement (INRAE), VetAgro Sup, Université Clermont Auvergne, Aurillac, France; 15 Institute for Biological Research Siniša Stanković, National Institute of the Republic of Serbia, University of Belgrade, Belgrade, Serbia; 16 Research Division Microbial Food Systems, Agroscope, Bern, Switzerland; 17 MED-Mediterranean Institute for Agriculture, Environment and Development and CHANGE-Global Change and Sustainability Institute, Departamento de Medicina Veterinária, Escola de Ciências e Tecnologia, Universidade de Évora, Évora, Portugal

**Keywords:** chemical hazards, fermented foods, food safety, foodborne outbreaks, foodborne pathogens, microbiological hazards, risks, risk assessment

## Abstract

**Background:**

Fermented foods (FF) are widely consumed across different dietary cultures and are increasingly recognized for their nutritional and functional properties. Although fermentation can improve food preservation and safety, there are still concerns about microbiological and chemical hazards. Given the growing consumption of FF, it is essential to conduct a systematic evaluation of their safety to support evidence-based risk-benefit assessments.

**Objectives:**

This review aimed to establish the safety of FF for human consumption and to identify the main microbiological and chemical hazards associated with these foods, including their occurrence, exposure and potential risks.

**Design:**

A systematic scoping review was conducted in accordance with a predefined protocol aligned with PRISMA principles. Relevant human studies were identified through searches of the PubMed, Scopus and Cochrane Library databases. The eligibility criteria were defined using a PECO framework and included all population groups and a broad range of FF across major food categories. Alcoholic beverages with an alcohol content above 1.25% were excluded. The outcomes included the occurrence of hazards, associated exposure and risk, and reported foodborne outbreaks.

**Results:**

Microbiological hazards were predominantly associated with pathogens such as *Salmonella*, pathogenic *Escherichia coli, Listeria monocytogenes, Campylobacter jejuni*, and *Clostridium botulinum*, with dairy and fermented meat products most frequently implicated in outbreaks. However, these risks were largely linked to contaminated raw materials, inadequate processing conditions, or post-process contamination rather than fermentation itself. Chemical hazards included mycotoxins, heavy metals, polycyclic aromatic hydrocarbons, and acrylamide. Most available risk metrics indicated low concern under typical consumption scenarios, although certain products, such as specific cheeses and cereal-based foods, showed higher exposure levels. Importantly, several chemical hazards were associated with raw material contamination or thermal processing rather than the fermentation process.

**Conclusion:**

Overall, the identified risks associated with FF are predominantly driven by contaminated raw materials, inadequate processing practices, and environmental factors, rather than by fermentation itself, supporting the safe integration of FF into modern diets when appropriate hygienic and technological conditions are ensured. These findings emphasize the importance of targeted risk management strategies focused on raw material quality, process control and harmonized risk assessment approaches.

## 1 Introduction

Fermented foods (FF) constitute a diverse class of foods and beverages whose production relies on the metabolic activity of microorganisms that transform raw substrates into products with distinct sensory, nutritional and functional properties. Fermented foods and beverages have been recently defined as “foods made through desired microbial growth and enzymatic conversions of food components” (1). They are ubiquitous across dietary traditions and, in many cultures, represent staples of daily diets. Historically, fermentation offered a robust strategy for preservation by lowering pH, producing antimicrobial metabolites and reducing water activity. Today, the same processes continue to contribute to safety and stability while also shaping organoleptic quality and nutritional value (2). In recent years, scientific and public interest in fermented foods has accelerated, driven by hypotheses about their capacity to deliver live microbes and fermentation-derived metabolites with potential benefits for human health, including modulation of the gut microbiome, enhancement of bioavailability of nutrients, and generation of bioactive compounds (3–6), as summarized in Table 1.

**Table 1:** Overview of the main recognized benefits associated with fermented foods across major food categories.

Food category	Main recognized benefits	References
Dairy (e.g., yogurt, kefir, cheese)	Enhanced digestibility, probiotic potential, calcium bioavailability	(1)
Fermented meat products	Extended shelf life, improved protein digestibility	(10)
Fermented vegetables (e.g., kimchi, sauerkraut)	Probiotic potential, enhanced vitamin content, antioxidant activity	(4)
Cereal-based (e.g., sourdough bread)	Improved digestibility, reduced antinutrients, lower glycaemic index	(6)
Fermented beverages (e.g., kombucha, kefir)	Probiotic potential, bioactive compound production	(7)

At the same time, it remains essential to systematically investigate their safety, recognizing that any food, fermented or not, may present hazards if the ingredients, processes or handling are inadequate (1). Observational and intervention studies, including broader syntheses, suggest that fermented foods can impact the gut microbiome structure and function, potentially affecting immune and metabolic readouts (7). However, causality and generalizability vary by product, context, and study design (1, 3, 8, 9). The ubiquity and growing popularity of fermented foods underscore the importance of evaluating their safety through transparent, evidence-based approaches.

From a process perspective, lactic acid fermentation, ethanol production, salt addition, drying and ripening steps typically produce combinations of hurdles (low pH, organic acids, ethanol, reduced water activity, bacteriocins) that suppress many pathogens and spoilage organisms (5, 10). This underlies the widespread use of fermentation as a preservation strategy across dairy, vegetable, cereals and legumes, meat and fish matrices (2, 5). Nevertheless, safety cannot be assumed, since vulnerability can arise at several points, most notably from contaminated raw materials, inadequate process control, cross-contamination after fermentation, or due to the formation and accumulation of certain metabolites under permissive conditions (1, 10–13). Furthermore, new risks may arise considering the development of novel FF (e. g. new strains, new matrices, etc…), particularly in light of the shift to plant-based foods as alternative protein sources (14, 15).

From a Food Safety perspective, microbiological hazards are central to the evaluation of fermented foods because they may enter with raw materials, persist when critical parameters are not adequately controlled, or be introduced post-process despite the protective hurdles created by fermentation (acidification, salt, reduced water activity, and microbial competition) (1, 10). At the surveillance level, European foodborne outbreak data are compiled by the European Food Safety Authority (EFSA) (16). However, the publicly available data present aggregated categories that do not always allow confirmation of the specific food product as the causal vehicle, reporting implicated food groups rather than verified outbreak sources. As such, potential raw materials for fermented foods, and in some cases fermented products themselves, may appear within these categories without permitting an easily definitive attribution, underscoring the need for a systematic appraisal of microbiological risks in this product class.

Regarding chemical risks in fermented foods, they fall into two broad groups: (i) contaminants in raw materials, e.g., mycotoxins and pesticide residues in cereals and heavy metals in fish, which (in this case)fermentation may reduce, redistribute or occasionally concentrate, but does not originate or explain their initial presence (12, 13); and, ii) compounds originated during fermentation, such as biogenic amines [e.g., histamine and tyramine produced by decarboxylase-positive microbes in high-protein matrices (17)], and acrylamide (a heat-induced, potentially carcinogenic contaminant on which fermentation can have a positive or negative effect, depending on many factors (18).

This scoping systematic review addresses two core questions: “Are fermented foods safe for human health?” and “Which microbiological and chemical hazards are associated with fermented foods, and what risks do they pose?”. Within PIMENTO - COST Action CA20128 “Promoting Innovation of ferMENTed fOods” Working Group 3, our specific aims were to: (i) characterize the occurrence of food hazards in fermented foods when linked to human consumption and reported illness; (ii) synthesize the reported risk of exposure to these hazards, considering product type and context of production; and, (iii) collate reported foodborne outbreaks attributed to fermented foods, including the extent of resulting disease (19).

Together, these objectives provide an evidence-based foundation to distinguish hazards arising from contaminated inputs or process deviations from those attributable to fermentation itself, and to inform proportionate risk management and surveillance priorities, particularly in the context of risk-benefit of fermented foods.

## 2 Methodology

This systematic scoping review was conducted according to a predefined protocol developed within the PIMENTO COST Action CA20128, Working Group 3 (WG3). This protocol followed the guidance of Muka et al. (20) and the PRISMA (Preferred Reporting Items for Systematic Reviews and Meta-Analyses) guidelines (21) and it was registered on the Open Science Framework: https://osf.io/hsakz/.

### 2.1 Research question

A systematic scoping review of the scientific literature on the safety of fermented foods aimed to answer two specific questions: (1) “Are fermented foods safe for human health?”; and (2) “Which microbiological and chemical hazards are associated with fermented foods, and what risks do they pose?”

### 2.2 Eligibility criteria

The eligibility criteria were established based on the PECO framework (22, 23). The population corresponded to the general population, including vulnerable groups (e.g., elderly, children, without any limitation on age, gender, health and physiological conditions (i.e., pregnant women, breastfeeding women, etc). The exposure consisted of the ingestion of any of the fermented foods contained in the PIMENTO search string for fermented foods across the following food groups: dairy, meat and fish, fruits and vegetables, beverages, legumes, cereals and grains. Alcoholic beverages with an alcohol content of more than 1.25% were excluded. Unless specified otherwise, no limits were set for duration or dosage of the ingested fermented food(s). Unless specified otherwise, studies investigating application of fermented foods other than for nutritional purpose (e.g., nasal or topical) were excluded. In addition, studies investigating probiotics were excluded unless the probiotic(s) were added at the beginning of the fermentation process and that there were indications from the literature that the probiotic strain(s) contribute(s) to the fermentation of the food matrix. Exposure, including any possible confounders such as prebiotic fibers or added bioactive compounds, were not included. The outcomes included reporting of (i) the occurrence (levels/magnitude; frequency) of food hazards in the defined fermented foods, when associated with human consumption and reported illness; (ii) the reported associated risk of exposure, taking into account the probability and severity; and (iii) the reported outbreaks that may occur and the extent of the diseases that occur. The comparison (i.e., absence of consumption of the fermented foods), was not considered.

### 2.3 Literature search strategy

Literature search for English written studies was conducted using PubMed, Scopus and Cochrane Library (Cochrane Reviews and Cochrane Trials) databases, covering the period from 01/01/1970 until 31/12/2024. A generic search string was developed by the Library of the University of Zurich in the context of the PIMENTO project, encompassing terms related to a broad scope of fermented foods across all major food groups, all types of human studies, and dietary intake outcomes. Safety-specific terms included food safety, contaminant, toxic, infection, and outbreak, among others. The full search string, including all terms and database-specific syntax, is provided in Supplementary Table S1. The predefined protocol underlying this systematic scoping review, including the complete search strategy, is publicly registered on the Open Science Framework: https://osf.io/hsakz/.

### 2.4 Study selection

Study selection was carried out using CADIMA-Central Access Database for Impact Monitoring and Assessment (https://www.cadima.info).

After deletion of duplicates, a consistency test training was performed in CADIMA before the study selection.

Then, study selection was conducted in two phases: (i) title and abstract screening; (ii) full text screening, both independently assessed by two reviewers. The inconsistencies found in both phases were solved by simple consensus between the two reviewers and mediated by the project leaders (R.A. and M.L.), whenever necessary.

### 2.5 Data extraction

Data were extracted independently by two reviewers and compiled by the project leaders. Standardized forms were used for microbiological and chemical hazards data extraction (Supplementary Table S2).

## 3 Results

### 3.1 Scoping results

**Figure 1:**
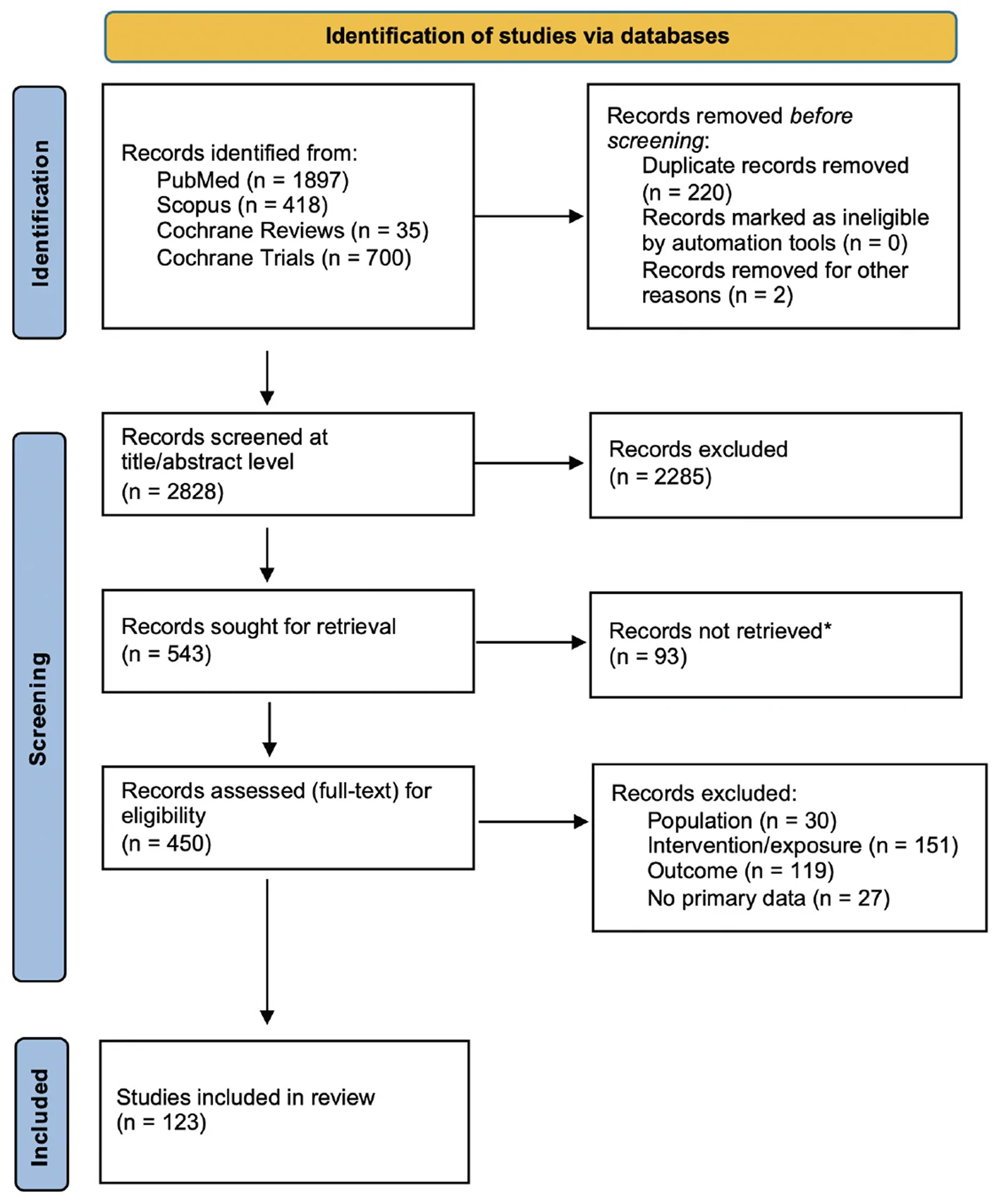
PRISMA chart illustrating the flowchart of studies to be included. Source: Page MJ et al. (21). *Full-text not available or text not in English.

Following literature search, papers were retrieved from three databases using the search string presented in Supplementary Table S1. After removal of duplicates, 2,828 papers were screened for eligibility based on their title and abstract. A total of 2,285 papers were excluded based on the title and abstract screening, while 543 papers were retained for full-text screening. The final selection included 123 studies that were kept for data extraction (Figure 1).

### 3.2 Microbiological hazards

The analysis of fermented foods safety incidents or outbreaks reveals several fermented foods associated with serious health risks due to microbiological contamination (Table 2).

**Table 2:** Summary of microbiological hazards from fermented foods.

Type of fermented foods	Fermented foods	Associated pathogens	# Hospitalizations/ # Deaths	References
Chocolate	Chocolate	Nontyphoidal *Salmonella*	-/0	(97)
	Chocolate	*Salmonella* Oranienburg	14/0	(98)
Dairy products	Artisan fresh cheese from raw cow's milk	*Toxoplasma gondii*	-/-	(37)
	Locally made cheese	*Clostridium botulinum*	4/-	(27)
	Cheese with “KochiKocha,” which was made from chili and butter	*Clostridium botulinum*	-/-	(25)
	Unpasteurised cheese	Verotoxin producing *Escherichia coli* (VTEC)	4/-	(99)
	Raw-milk cheese	*Salmonella* Dublin	-/0	(100)
	A pecorino cheese made with unpasteurized sheep milk	enteroaggregative *Escherichia coli* (EAEC) serotype O92: H33	0/0	(101)
	Hard cheeses	*Mycobacterium avium* complex (MAC)	-/-	(102)
	Mexican-style soft cheese	*Listeria monocytogenes*	-/-	(103)
	Unpasteurised bovine milk cheese	*Streptococcus equi zooepidemicus*	-/-	(104)
	Cheddar cheese	*Salmonella* Heidelberg	-/-	(105)
	Raw milk cheese	*Escherichia coli* O157:H7	-/0	(106)
	Processed white cheese	*Streptococcus* T type 8/25/Imp19	-/0	(107)
	Unpasteurized goats and sheep milk or cheese	*Brucella melitensis Brucella suis* bv 1	-/0	(108)
	Brie cheese	*Listeria monocytogenes*	-/-	(109)
	Cheese	*Campylobacter jejuni*	44/-	(110)
	Unpasteurized Goat cheese	*Salmonella* Paratyphi B	-/1	(111)
	Fresh pasteurized milk cheese	*Shigella sonnei*	-/-	(112)
	Gorgonzola cheese	*Listeria monocytogenes* serotype 1/2b	1/1	(113)
	Latin-style fresh cheese	*Listeria monocytogenes*	2/0	(114)
	Mozzarella cheese	*Salmonella* Javiana *Salmonella* Oranienburg	-/-	(115)
	Raw cheese	*Brucella melitensis*	-/-	(116)
	Leeks in cheese sauce	*Clostridium perfringens*	1/-	(117)
	Cheese	*Clostridium botulinum* Type A	23/1	(28)
	Mexican-style cheese	*Listeria monocytogenes*	13/5	(118)
	Unpasteurized Mexican-style aged cheese (Cotija cheese)	Multidrug-Resistant *Salmonella* Newport	36/0	(119)
	Soft unpasteurized cow's milk cheese (imported Irish cheese)	*Salmonella* Dublin	3/0	(120)
	Soft cheese made from raw cow's milk	*Salmonella* Montevideo	5/2	(121)
	Yogurt-based relish used in kebabs	*Salmonella* Typhimurium DT170	6/0	(122)
	Cheese (along with tuna fish and chicken salad, suspected as potential vehicles of infection)	*Listeria monocytogenes* serotype 4b	23/5	(123)
	Yogurt	*Salmonella* Typhi	74/-	(124)
	Cheese	*Brucella* spp.	-/-	(125)
	Camembert cheese	*Listeria monocytogenes*	0/0	(126)
	Fresh Mexican style cheese	*Salmonella* Typhimurium	14/-	(127)
	Cantal cheese	*Salmonella enterica*	-/-	(128)
Cereal products	Bread	Norovirus GII	0/0	(32)
	Hamburguer bun	*Salmonella* Thompson	9/0	(129)
	Bread	norovirus	-/-	(33)
Non-alcoholic beverages	Bushera	*Salmonella* Typhi	-/-	(130)
Vegetable products	Kimchi cabbage	Enterotoxigenic *Escherichia coli* (ETEC) O6	0/-	(131)
	Kimchi	*Escherichia coli* O169	0/0	(132)
	Kimchi	Norovirus GI.4	0/0	(34)
	Fermented black beans (douchi)	*Bacillus cereus*	-/-	(24)
	Natto	*Bacillus subtilis*	1/0	(133)
	Natto	*Bacillus subtilis* var. *natto*	1/0	(134)
Meat and/or fish products	Cold sliced salami	not identified	-/-	(135)
Meat and/or fish products	Salt-Cured Fish	*Clostridium botulinum* type E	2/0	(26)
Meat and/or fish products	Fermented oysters (“eorigul-jeot”)	Norovirus (NoV) Genogroups GII.4, GII.11, GII.14	-/0	(31)
Meat and/or fish products	Cured pork meat	Hepatitis E virus	-/-	(35)
Meat and/or fish products	Pork salami	*Salmonella* Typhimurium DT104A	-/0	(136)
Meat and/or fish products	Dry fermented salami, raw beef, raw beef suet, and raw pork were combined	*Escherichia coli* O157:H7	-/0	(137)
Meat and/or fish products	Genoa Salami	*Escherichia coli* O157:H7	14/0	(138)
Meat and/or fish products	Salami	*Listeria monocytogenes*	-/-	(109)
Meat and/or fish products	Salami	*Salmonella* Montevideo	52/0	(139)
Meat and/or fish products	Fermented sausage	*Escherichia coli* O157:H7	13/0	(140)
Meat and/or fish products	Cinkrugan (fermented goat meat)	*Clostridium botulinum* Type B	3/0	(29)
Meat and/or fish products	Dried pork sausage (“longaniza de Pascua”)	*Salmonella* Typhimurium (monophasic and biphasic) *Salmonella* Derby	-/0	(141)
Meat and/or fish products	Organic fermented beef sausage	Shiga toxin-producing *Escherichia coli* (STEC) O26:H11	0/0	(30)
Meat and/or fish products	Fermented raw pork sausage (spreadable “frische Mettwurst”)	*Salmonella* Goldcoast	-/0	(142)
Meat and/or fish products	Duck prosciutto (cured duck meat)	*Salmonella* Typhimurium PT9	7/0	(143)
Meat and/or fish products	Salami-type sausage and highly seasoned pork sausage	*Trichinella britovi*	-/0	(36)
Meat and/or fish products	Salami	*Salmonella* Senftenberg	-/-	(144)
Meat and/or fish products	Pork dry-fermented salami	*Escherichia coli* 0157	2/0	(145)
Meat and/or fish products	Cured mutton sausages	*Escherichia coli* O103:H25	12/-	(146)
Meat and/or fish products	Salami	*Salmonella* Typhimurium	21/-	(147)

- not reported.

For the purpose of this review, the identified microbiological hazards have been considered according to the most plausible point of entry in the production chain: (i) hazards entering via contaminated raw materials; (ii) hazards resulting from inadequate process control during fermentation; and (iii) hazards introduced post-fermentation through cross-contamination or improper handling. This categorization is intended to avoid implicit attribution of these risks to fermentation as a preservation technology.

Bacteria were the most commonly identified microbiological hazards, with *Salmonella, Escherichia coli* and other enterobacteria, as well as *Campylobacter jejuni* and *Clostridium botulinum* being the most frequently reported (highest number of reported cases), while *Listeria monocytogenes* shows up as the most lethal.

Concerning toxin-producing pathogens, three *B. cereus* strains (cereulide and enterotoxins) (24), different *Clostridium botulinum* strains (toxins types A, B and E) (25–29), and one STEC strain (Shiga toxin) have been reported (30).

Regarding viruses, only hepatitis E virus and noroviruses have been reported associated to outbreaks (31–35). *Toxoplasma gondii* and *Trichinella britovi* were the two parasites-that have been reported to be involved in foodborne outbreaks (36, 37).

Cheese and yogurt are the fermented foods most frequently associated with foodborne outbreaks of microbiological origin, with contamination most frequently traceable to raw milk quality, inadequate pasteurization, or post-process handling rather than to the fermentation process itself. Thus, these food products are the FF that pose the greatest concerns in terms of food safety. Fermented dairy products are also the FF type that have shown the highest prevalence of consumption (38).

Several epidemiological investigations have highlighted that certain fermented foods have been implicated in severe foodborne outbreaks resulting in a notable number of hospitalizations and, in some cases, fatalities. Among these, fermented dairy products appear most frequently associated with significant public health concerns, with contamination predominantly linked to the use of raw or inadequately pasteurized milk, poor hygienic conditions during production, or post-process cross-contamination.

In particular, within the dairy sector, yogurt has been linked to 74 hospitalizations, primarily associated with *Salmonella* Typhi contamination. However, cheese products have been recurrently implicated in outbreaks involving different pathogenic species. For example, cheese contaminated with *C. jejuni* caused 44 hospitalizations, while unpasteurized Mexican-style aged cheese (Cotija) was responsible for 36 hospitalizations associated with multidrug-resistant *Salmonella* Newport. Another serious outbreak was linked to a salad containing cheese, tuna fish and chicken, which resulted in 23 hospitalizations due to *L. monocytogenes* serotype 4b.

Among the food categories, fermented meat products, such as salami, accounted for 52 and 21 hospitalizations related to *Salmonella* Montevideo and *Salmonella enterica* Typhimurium, respectively.

Deaths have also been reported following the consumption of cheese-based products. Both cheese with tuna fish and chicken salad and Mexican-style cheese were responsible for five deaths each, in both cases due to *L. monocytogenes* infections. Soft cheese made from raw cow's milk was linked to two deaths caused by *Salmonella* Montevideo, while isolated fatal cases involved *C. botulinum* type A in a generic cheese product and *L. monocytogenes* serotype 1/2b in Gorgonzola cheese.

Importantly, from the analysis of the retrieved studies, the identified microbiological risks rarely emerge from the fermentation process itself. In the majority of cases, hazards were attributable to contaminated raw materials, inadequate process control, or post-fermentation contamination, underscoring that fermentation, when properly conducted, remains a robust preservation strategy.

These findings collectively highlight that the microbiological risks associated with fermented foods are predominantly attributable to failures at specific points in the production chain, e.g., raw material quality, process control, and post-fermentation handling, rather than to fermentation itself. This underscores the importance of rigorous hygiene standards, adequate pasteurization where applicable, and systematic monitoring of key pathogens such as *Listeria monocytogenes, Salmonella* spp., and *Clostridium botulinum* in fermented food production.

### 3.3 Chemical hazards

The analysis of fermented food safety incidents or outbreaks suggests various fermented foods associated with relevant health problems caused by chemical hazards, such as mycotoxins, heavy metals, polycyclic aromatic hydrocarbons (PAHs), and acrylamide. Importantly, for most of these contaminants, the available evidence indicates that their presence in fermented foods reflects upstream contamination of raw materials or co-occurring thermal processing steps, rather than the fermentation process itself.

#### 3.3.1 Mycotoxins

Across the extracted records (Table 3), mycotoxin exposure associated with fermented foods clustered around cereal-based products (bread and bakery items) and fermented dairy (yogurt, kefir, fermented milk, cheese), with additional contributions from coffee. Table 3 summarizes, for each food type and toxin, the reported intake and risk metrics used for risk characterization.

**Table 3:** Summary of dietary exposure and risk characterization of mycotoxins from fermented foods.

Type of fermented foods	Mycotoxins	Mean estimated intake (ng/kg bodyweight/day)	Risk characterization	References
Cereal products	Aflatoxin B1	0.002–0.02	max HQ 0.0011 min MOE 21,193.14 max LCR 0.0016	(39)
	Aflatoxin B2	0.080	NR	(40)
	Aflatoxin G1	0.080	NR	(40)
	Aflatoxin G2	0.080	NR	(40)
	Aflatoxins (total)	0.119	NR	(41)
	Ochratoxin A	0.172	NR	(40)
		0.01 – 1.18	max HQ 0.0653 min MOE 12,331.48	(39)
	Deoxynivalenol (DON)	332.18	max HQ 0.0415	(39)
	Deoxynivalenol (DON) and metabolites (3-Ac-DON, 15-Ac-DON)	DON: 226.3 3-Ac-DON: 5.45 15-Ac-DON: 4.82	NR	(40)
	Nivalenol	13.29	NR	(40)
	Zearalenone	0.706	NR	(41)
		8.03	NR	(40)
	Fumonisins B1 (FB1) and B2 (FB2)	FB1: 13.49; FB2: 3.47	NR	(40)
		2.936	NR	(41)
Dairy products	Aflatoxin M1	0.06	max HQ 0.33 min MOE 14,561 max LCR 0.00038	(42)
		0.003	max HQ 0.174	(39)
		0.031	max HQ 0.12 min MOE 127,389	(43)
		0.04	max HQ 0.19	(44)
		Range 0.06 - 0.09	min MOE 6,482 max LCR 0.0005	(45)
Dairy products	Aflatoxin M1	Range 0.06 - 0.07	max HQ 0.3496	(39)
		Range 0.00026 - 0.29	max HQ 0.022 min MOE 40,000	(42)
Coffee	Ochratoxin A	0.00037	NR	(46)

For each food category and mycotoxin, the table reports intake (median and range by unit) and risk metrics. HQ, hazard quotient; MOE, margin of exposure; LCR, lifetime cancer risk). HQ > 1 indicates potential non-cancer concern; for genotoxic carcinogens (e.g., aflatoxins, OTA), an MOE ≥ 10,000 is generally considered of low concern, whereas MOE < 10,000 indicates potential concern; typical population-level LCR screening bands are within the 10^−6^−10^−5^ range. NR, not reported.

For bread and bakery, aflatoxin B1 (AFB1) intakes of 0.002-0.02 ng/kg bw/day corresponded to a maximum HQ of 0.0011, a minimum MOE of 21193, and a maximum LCR of 0.0016 (39). Additional aflatoxins (AFB2, AFG1, AFG2) were also reported at 0.080 ng/kg bw/day without risk characterization (40, 41). Ochratoxin A (OTA) ranged from 0.01–1.18 ng/kg bw/day (max HQ 0.0653; min MOE 12331) (39, 40). For deoxynivalenol (DON), a worst-case intake of 332.18 ng/kg bw/day yielded an HQ 0.0415 (39, 40). Reported intakes for nivalenol (13.29 ng/kg bw/day), zearalenone (0.706 and 8.03 ng/kg bw/day) and fumonisins (FB1 13.49; FB2 3.47 ng/kg bw/day) lacked risk metrics, limiting formal appraisal beyond risk magnitude (40, 41).

In dairy, all the available data concerned aflatoxin M1 (AFM1). For cheese, intakes from 0.003–0.06 ng/kg bw/day produced maximum HQs of 0.174–0.33, minimum MOEs of 14,561–127,389, and maximum LCRs up to 0.00038 (39, 42–44). One study reported at 0.06–0.09 ng/kg bw/day and a minimum MOE of 6,482 and a maximum LCR of 0.0005, indicating potential health concern (45). For yogurt/kefir and fermented milk, AFM1 ranged 0.06–0.07 ng/kg bw/day (max HQ 0.3496) and 0.00026–0.29 ng/kg bw/day (max HQ 0.022; min MOE 40,000), consistent with low concern (39, 42). OTA intake through coffee consumption (0.00037 ng/kg bw/day) showed no risk metrics to characterize the associated risk (40).

Overall, wherever risk metrics were available, HQs were < 1 and MOEs were generally ≥ 10,000, except for cheese/cream cases, generally suggesting low concern of mycotoxins under the specific consumption scenarios and populations assessed, for most fermented foods assessed.

However, the absence of risk metrics, particularly for zearalenone, fumonisin, and nivalenol constrains definitive risk characterization (39–46). It should be noted that fermentation itself does not generate mycotoxins; their presence in fermented products reflects fungal contamination of raw materials prior to processing. While some evidence suggests that certain fermentation processes may contribute to mycotoxin reduction through microbial binding or biotransformation, these effects are variable and cannot be relied upon as a consistent mitigation strategy.

#### 3.3.2 Heavy metals

Table 4 summarizes the concentrations in products (μg/kg or mg/kg) and/or estimated dietary intakes (μg/kg bw/day) and risk metrics (THQ/HI/MOE). The results are organized by product group (cereal-based breads/bakery; fermented dairy including cheese/cream, yogurt/kefir/fermented milk; and coffee/chocolate).

**Table 4:** Summary of dietary exposure and risk characterization of heavy metals from fermented foods.

Type of fermented foods	Fermented foods	Heavy metals	Product concentration	Mean estimated intake	Risk characterization - RC/Hazard index - HI/Target hazard quotient – THQ/Generalized Additive Model GAM	References
Cereal products	Multi-grain bread, whole meal bread, whole wheat bread, rye bread, and white bread:	Mn, Cu, Ni, Pb, As, Cr, Co, Cd, and Hg	Mn: 19,084–28,151; Cu: 3,139–4,377; Ni: 260–710; Pb: 20.1–56.3; As: 13.4–22.0; Cr: 49.0–127; Co: 7.20–25.3; Cd: 11.0–23.6; and Hg: 0.13–0.41 μg/kg	Mn: 44.0, Cu: 6.62, Ni: 0.69, Pb: 0.15, As: 0.06, Cr: 0.04, Co: 0.03, Cd: 0.03, and Hg: < 0.00 μg/kg bw/day	THQ < 1; HI (1.37 - 1.71)	(148)
	Bread	Methylmercury	1.4 mg/loaf of bread	/		(48, 149)
	Bread	Pb, Ni	Pb: 13.64 μg/kg; Ni: 194 μg/kg	Pb: 0.0267 μg/kg/day; Ni: 0.3802 μg/kg/day	MOE 18.7	(52)
	Bread	As, Cd, Cr, Ni, Pb	As 0.11–1.31; Cd 0.13–0.2; Cr 0.056–1.44; Ni 0.61–1.2; Pb 0.054–0.36 mg/kg	/	HI < 1 and HQ < 1 (adults and children)	(150)
	Bread	Fe, Zn, Cr, Cd, Pb, As, Ni and Sr	Fe: 129–316; Zn: 18.14–36.14; Cr: 0.049–1.39; Cd: 0.13–0.19; Pb: 0.054–0.41; As: 0.11–1.29; Ni: 0.61–1.2; Sr: 1.64–9.89 ppm	/	GAM: Pb: 0.69(SE: 0.015–43.92); As: 3.58(SE: 0.28–12.36); Sr:11.88681 (0.285–41.70) Cd: 0.04190 (0.017–2.338)	(49)
					GAM: Pb: 0.14(SE: 0.008–16.01); As: 0.24(SE: 0.03–7.8); Sr:2.6582 (0.166–16.01) Cd: 0.03310 (0.015–2.103)	
Dairy products	Processed dairy products	Ti	0.12 (±0.17) mg/kg	/	NR	(51)
	Sour cream	Pb	Pb: 1.98 μg/kg; Ni: 48 μg/kg	Pb: 0.0004 μg/kg/day; Ni: 0.0109 μg/kg/day	MOE 1111.6	(52)
		Cheese	Pb: 52.24 μg/kg; Ni: 48 μg/kg	Pb: 0.0087 μg/kg/day; Ni: 0.0214 μg/kg/day	MOE 57.4	
		Cheese curd	Pb: 183.28 μg/kg Ni: 135 μg/kg	Pb: 0.2327 μg/kg/day; Ni: 0.1714 μg/kg/day	MOE 2.15	
	Sheep cheese	Sb	0.22 ±0.15 mg/kg	0.0003/0.0001 mg/day/100 g	THQ preschool children: 2.5; adults: 0.79	(50)
		As	0.73 ± 0.29 mg/kg	0.0010/0.0033 mg/day/100 g	THQ preschool children: 11.06; adults: 3.48	
		Ba	1.26 ± 0.58 mg/kg	0.0018/0.0057 mg/day/100 g	THQ preschool children: 0.03; adults: 0.01	
		Cr	0.04 ± 0.01 mg/kg	0.0001/0.0001 mg/day/100 g	THQ preschool children: 0.06; adults: 0.02	
		Cu	0.26 ± 0.08 mg/kg	/	THQ preschool children: 0.0; adults: 0.01	
		Fe	6.57 ± 0.84 mg/kg	/	THQ preschool children: 0.0; adults: 0.01	
		Pb	0.13 ± 0.09 mg/kg	0.0002/0.0006 mg/day/100 g	THQ preschool children: 0.17; adults: 0.05	
		Li	0.07 ± 0.03 mg/kg	0.0001/0.0003 mg/day/100 g	THQ preschool children: 0.09; adults: 0.03	
		Mo	0.06 ± 0.05 mg/kg	0.0001/0.0003 mg/day/100 g	THQ preschool children: 0.05; adults: 0.02	
		Ni	0.70 ± 0.52 mg/kg	0.0010/0.0032 mg/day/100 g	THQ preschool children: 0.16; adults: 0.05	
		Sr	4.96 ± 0.74 mg/kg	0.0071/0.0226 mg/day/100 g	THQ preschool children: 0.04; adults: 0.01	
Dairy products	Fermented milk	Hg, Pb, Cd, Cr, As, Zn, Cu, Fe, Mn, Ni	Hg: 0–26; Pb: 0–35.01; Cd: 0–305; Cr: 0–433; As: 0–17; Zn: 2,893–28,980; Cu: 31.09–1,133; Fe:341.83–8320; Mn:22.67–793; Ni: 0–81.9 μg/kg	/	THQ < 1; HI < 1 THQ < 1; HI < 1	(53)
	Matsun	Pb, Ni	Pb: 0.84 μg/kg; Ni: 19 μg/kg	Pb: 0.0003 μg/kg/day; Ni: 0.0075 μg/kg/day	MOE 1513.8	(52)
	Yogurt	Ti	0.47 (±0.46) mg/kg	/	NR	(51)
Coffee & Chocolate	Chocolate	As	^*^	^*^	NR	(47)
	Coffee	Pb	Pb: 192.56 μg/kg; Ni: 142 μg/kg	Pb: 0.1398 μg/kg/day; Ni: 0.1031 μg/kg/day	MOE 3.6	(52)
	Coffee	Cd	Range: 0.002–0.100 mg/kg	/	THQ < 1	(54)
		Cu	Range: 0.507–10.527 mg/kg	/		
		Pb	Range: 0.033–0.695 mg/kg	/		
		Ni	Range: 0.952–1.511 mg/kg	/		
		Zn	Range: 4.472–26.109 mg/kg	/		

THQ, Target hazard quotient; HI, hazard index; MOE, -margin of exposure. ^*^Arsenic poisoning through chocolate.

In multigrain, wholemeal (or whole wheat), rye, and white breads, several metallic elements are present, including manganese (Mn), copper (Cu), nickel (Ni), lead (Pb), arsenic (As), chromium (Cr), cobalt (Co), cadmium (Cd), and mercury (Hg). The average daily intake of these metals is primarily dominated by manganese at 44.0 μg/kg body weight and copper at 6.62 μg/kg body weight. Concentrations of these metals in bakery products can vary significantly, e.g., manganese levels range from 19,084 to 28.151 μg/kg, copper from 3.139 to 4.377 μg/kg, nickel from 260 to 710 μg/kg, and lead from 20.1 to 56.3 μg/kg. While individual Target Hazard Quotients (THQs) are less than 1, the cumulative Hazard Index (HI) reaches between 1.37 and 1.71. This suggests a potential cumulative effect, even though single-metal assessments seem acceptable. Additional analyses of bread products indicated lead and nickel contributions with a reported Margin of Exposure (MOE) of 18.7, which may represent a particular concern. Regarding the methylmercury and bread consumption, the studies reported an outbreak occurring in the 1970′s in Iran, using wheat flour treated with a methylmercury fungicide (47, 48). Together, these findings highlight the importance of product formulation (such as whole grain or bran inclusions), and the specific types of metals present in determining the risk outcomes associated with these bakery products.

In Neisi, Farhadi, Cheraghian et al. (49), Generalized Additive Models (GAM) were employed for advanced exposure assessment in healthy and patient groups, providing probabilistic insights into the exposure-risk relationships between heavy metals in food and cardiovascular diseases by statistically comparing the mean concentrations of heavy metals in urine between the patient group and the healthy group. GAM analysis revealed that the levels of Sr and Cd in bread exceeded Iran's standard limit (49). There was a significant correlation (*p*-value < 0.05) between urinary heavy metal levels in individuals with heart disease and those in the healthy group, excluding Ni.

A detailed profile of sheep cheese reveals several concerning elements, with Target Hazard Quotients (THQs) calculated for both preschool children and adults. The most notable finding is arsenic (As), with THQ values of 11.06 for preschool children and 3.48 for adults, reflecting a mean product level of 0.73 ± 0.29 mg/kg. Antimony (Sb) also presented significant values, with a THQ of 2.5 for preschool children and 0.79 for adults, and a mean level of 0.22 ± 0.15 mg/kg. Other elements such as barium (Ba), chromium (Cr), copper (Cu), iron (Fe), lead (Pb), lithium (Li), molybdenum (Mo), nickel (Ni), and strontium (Sr) showed THQs well below 1 across all groups. These findings highlight that cheese type and origin are crucial factors, with exposure in young children being the most critical concern (50).

In processed dairy products, titanium (Ti) was detected at 0.12 ± 0.17 mg/kg, although risk levels were not reported (51). Specific entries indicate lead and nickel in sour cream (Pb 1.98 μg/kg, Ni 48 μg/kg), which have a relatively high MOE of 1,111.6, considered by the authors as of low concern. Conversely, cheese had lead levels of 52.24 μg/kg and nickel at 48 μg/kg, with an MOE of 57.4. Cheese curd had even higher lead levels at 183.28 μg/kg and nickel at 135 μg/kg, yielding an MOE of 2.15, indicating the narrowest margin of safety among dairy products concerning lead exposure (52). Based on the available evidence, most cheese and cream varieties appear to pose low concern under the assessed consumption scenarios, but certain products, like sheep cheese and specific curds, exhibit elevated lead and arsenic levels that necessitate targeted sourcing controls and verification testing, particularly for items marketed to children.

In the realm of fermented milk, a wide array of metals (including mercury (Hg), lead (Pb), cadmium (Cd), chromium (Cr), arsenic (As), zinc (Zn), copper (Cu), iron (Fe), manganese (Mn), and nickel (Ni)) showed ranges such as Pb from 0 to 35.01 μg/kg, Cd from 0 to 305 μg/kg, Cr from 0 to 433 μg/kg, and As from 0 to 17 μg/kg. All assessed metals had a THQ of less than 1, and the Hazard Index (HI) was below 1 overall, indicating a low risk under typical intake scenarios (53). For the traditional Armenian fermented milk Matsun, the estimated intakes of lead (0.84 μg/kg) and nickel (19 μg/kg) resulted in a relatively high MOE value of 1,513.8, considered by the authors as of low concern (52).

Yogurt showed titanium levels of 0.47 ± 0.46 mg/kg, but risk characterization was not provided (47).

For coffee, various surveys report cadmium levels ranging from 0.002 to 0.100 mg/kg, copper from 0.507 to 10.527 mg/kg, lead from 0.033 to 0.695 mg/kg, nickel from 0.952 to 1.511 mg/kg, and zinc from 4.472 to 26.109 mg/kg (54). A separate dataset estimates lead intake at 0.1398 μg/kg body weight per day (with nickel at 0.1031 μg/kg body weight per day), which presents a relatively tighter MOE compared to dairy products (52). In individual case reports, chocolate was identified as a potential source of arsenic. In one report, arsenic biomonitoring in exposed individuals detected concentrations of 0.3–0.4 mL/L in blood and 5.3–5.8 mL/L in urine, and high maternal exposure was linked to fetal mortality (55).

While direct comparisons across studies are limited due to context and unit variations, these findings suggest that coffee (for lead exposure) and certain cocoa/chocolate products (for arsenic exposure) can be significant contributors to specific consumption patterns.

The heterogeneity in sampling frames, origins, and analytical methods (and occasional non-reported risk fields) restricts strict cross-study comparability. Furthermore, biomonitoring entries (e.g., arsenic levels in chocolate consumers) reflect specific contexts that may not be generalized. Future studies should aim to harmonize consumption scenarios, incorporate distributional intake reporting, and prioritize the investigation of speciation (e.g., inorganic arsenic) where feasible.

#### 3.3.3 Polycyclic aromatic hydrocarbons (PAH)s

Across cereal-based fermented foods, PAH intakes and, when available, risks were described exclusively for bread (Table 5). PAHs are not generated by fermentation and their occurrence in fermented foods is attributable to co-occurring thermal processing steps, particularly smoking and baking, rather than to microbial activity. Benzo[a]pyrene (BaP) represented the main quantified compound, with reported mean daily intakes ranging from 48.1 ± 20.4 ng/day to 4 × 10^−5^−1.02 μg/kg bw/day. Carcinogenic risk values indicated that bread consumers were exposed to moderate or high carcinogenic risk levels through the intake of PAHs (56). Grouped PAH fractions (PAH4, PAH8, PAH16) were also reported but lacked quantitative intake or risk data. Overall, available results suggest that, although bread constitutes a relevant contributor to PAH dietary exposure, the limited number of studies and incomplete reporting of risk metrics prevent a comprehensive risk assessment.

**Table 5:** Summary of dietary exposure and risk characterization of PAHs from fermented foods.

Type of Fermented foods	PAHs	Mean estimated intake (ng/kg bw/day)	Risk characterization	References
Bread	Benzo(a)pyrene (BaP)	48.1 ± 20.4 ng/day	NR	(151)
	Benzo(a)pyrene (BaP)	4 × 10^−5^ to 1.02 μg/kg/day	moderate or high carcinogenic risks (CR > 10^−6^)	(56)
	PAH 4: the combined levels of benzo[a]pyrene (BaP), chrysene (CHR), benz[a]anthracene (BaA), and benzo[b]fluoranthene (BbF)	NR (mean concentration of PAH4 in bread samples from various countries worldwide varied between not detected to 3.45 μg/kg)	NR	(56)
	PAH 8: benz[a]anthracene, benzo[b]fluoranthene, benzo[k]fluoranthene, benzo[ghi]perylene, benzo[a]pyrene, chrysene, dibenz[a,h]anthracene, and indeno[1,2,3-cd]pyrene	NR (mean concentration of PAH8 in bread samples from various countries worldwide varied between not detected to 14 μg/kg)	NR	(56)
	PAH16: acenaphthene, acenaphthylene, anthracene, fluoranthene, fluorene, naphthalene, phenanthrene, pyrene, benz[a]anthracene, benzo[b]fluoranthene, benzo[k]fluoranthene, benzo[ghi]perylene, benzo[a]pyrene, chrysene, dibenz[a,h]anthracene, and indeno[1,2,3-cd]pyrene	NR (mean concentration of PAH16 in bread samples from various countries worldwide varied between 1.06 and 374.8 μg/kg)	NR	(56)

For each food category and PAH, the table reports intake (median and range by unit) and risk metrics (max HQ, min MOE, max LCR). HQ > 1 indicates potential non-cancer concern; for genotoxic carcinogens (e.g., benzo[a]pyrene), an MOE ≥ 10,000 is generally considered of low concern, whereas MOE < 10,000 indicates potential concern; typical population-level lifetime cancer risk (LCR) screening bands are 10^−6^−10^−5^. NR, not reported.

#### 3.3.4 Acrylamide

Acrylamide, which has recently been the subject of intense research due to its association with increased carcinogenic, neurotoxic and genotoxic risks from exposure to heat-treated foods, contributes to the risk of fermented foods from the consumption of bread, coffee, and tea. Acrylamide formation is driven by Maillard chemistry during high-temperature processing steps such as baking and roasting, and is not a product of fermentation. However, fermentation may indirectly modulate acrylamide formation by altering precursor availability and pH. Table 6 summarizes the reported acrylamide intake from fermented foods (bread, brewed coffee, tea).

**Table 6:** Summary of dietary exposure and risk characterization of acrylamide from fermented foods.

Type of fermented foods	Fermented foods	Mean estimated intake (μg/kg bw/day)	References
Bread & Bakery (cereal-based)	White bread	0.0340	(57)
	Bread (age 1–6)	0.2325	(58)
	Bread (age 7–19)	0.2480	(58)
	Bread (age 19–96)	0.1617	(58)
	Bread (age 1–96)	0.1935	(58)
Coffee	Coffee	0.1700	(57)
	Brewed coffee	0.0110	(59)
	Coffee beverage	0.0016	(59)
	Coffee (age 7–19)	0.0062	(58)
	Coffee (age 19–96)	0.0891	(58)
	Coffee (age 1–96)	0.0817	(58)
Tea	Green tea and oolong tea	0.0042	(59)
	Barley tea, bottled	0.0018	(59)
	Barley tea, home prepared	0.0018	(59)
	Rosted green tea	0.0018	(59)

NR, not reported.

Bellicha et al. (57) reported that the main contributors to acrylamide intake were coffee (mean ± SD: 10.4 ± 13.6 μg/d), potato fries and chips (mean ± SD: 10.1 ± 16.3 μg/d), pastries and cakes (mean ± SD: 3.1 ± 3.3 μg/d), and bread (mean ± SD: 2.1 ± 1.6 μg/d). Overall, food groups that can be considered fermented foods (coffee, bread, pastries, and cakes) contributed approximately 56% of total intake. A borderline significant positive association was observed between dietary acrylamide exposure and breast cancer risk overall. The association was observed more specifically in premenopausal females. However, data on the association between acrylamide intake from fermented foods (bread, pastries, coffee) and breast cancer are not specifically available in this study. Similar findings were observed in a Polish study (58): the main sources of dietary acrylamide are bread (45%), French fries and potato crisps (23%), and roasted coffee (19%). Acrylamide exposure from bread varies between 0.16 and 0.25 μg/kg body weight/day, and from coffee approximately 0.08 μg/kg body weight/day for total age group. The observations are similar in other European countries. The risk characterization of acrylamide intake from fermented foods could not be extracted from these data.

Data from a Japanese study (59) revealed that coffee, along with potato and vegetable cooked at high temperature, contribute significantly to long term dietary acrylamide exposure. The calculated MOE values for the median and 95th percentile of probable long-term average dietary acrylamide exposure using the non-carcinogenic endpoint were 2,792 and 1,648, which indicates that neoplastic risk cannot be excluded. Separate data for fermented foods cannot be extracted from this study.

According to the study by McCullough et al. (60), based on the nutrition cohort of 151,337 participants, dietary acrylamide is not associated with renal cell cancer (RCC) risk. As the main contributors of acrylamide in American populations were identified French fries (23% of intake), coffee (15%) and bread (10%). Acrylamide intake was not associated with RCC among men, women, or both combined after adjusting for age and sex or in multivariable analyses.

#### 3.3.5 Other chemical hazards

The analyzed studies of various hazardous organic and inorganic compounds identified in fermented foods reported the presence of multiple contaminants, including dioxins, bisphenol A, bromates, nitrosamines, alkaloids, ultraviolet filters, furan, and trace elements such as arsenic, among others. Table 7 summarizes, for each food type and hazardous organic and inorganic compounds, the reported intake and risk metrics used for risk characterization.

**Table 7:** Summary of dietary exposure and risk characterization of other hazardous organic and inorganic compounds from fermented foods.

Type of fermented food		Hazardous compounds		Mean estimated intake/Mean concentration	Risk characterization	References
Dairy	Not specified	Residues of antibiotics:	Ampicillin	Range 2.44 to 3.89 μg/L	NR	(62)
	Not specified		Tetracycline	Range 54.13 to 220.3 μg/L	NR	(62)
	Not specified		Oxytetracycline	Range 41.55 to 160.7 μg/L	NR	(62)
	Not specified		Amoxicillin	Range 3.11 to 5.5 μg/L,	NR	(62)
	Cheese and yogurt	Dioxin-like contaminants		Range 204–233 g/day	NR	(152)
	Yogurt			53.7 g/day	NR	(153)
	Dairy dessert			7.7 g/day	NR	(153)
	Cheese			37 g/day	NR	(153)
	Cheese	PCDD, PCDF, and PCB Dioxin		4.88 pg WHO-TEQ/kg bw/day	the maximum WHO TDI of 4 pg TEQ/kg bw/day	(61)
	Not specified			36.9–252 pg/day	NR	(154)
	Cheese and yogurt	Bisphenol A (BPA)		521.0 ng/mL−640 ng/mL	NR	(155)
Bread & bakery (cereal-based)	French bread	Potassium bromate		31.83 mg/kg/day	cHQ: 1,591.50/aHQ: 2,387,125/HR: 190,980.00	(63)
	Milk bread			36.89 mg/kg/day	cHQ: 31,844.89/aHQ: 2,537,125/HR: 221,387.10	(63)
	Simple bread			49.19 mg/kg/day	cHQ: 2,459.36/aHQ: 2,287,125/HR: 295,122.90	(63)
	Wheat bread			11.56 mg/kg/day	cHQ: 577.93/aHQ: 774,625/HR: 69,351.43	(63)
	Local bread			5.56 mg/kg/day	cHQ: 277.93/aHQ: 174,625/HR: 33,351.43	(63)
	Not specified			0.0000525 mg/kg/day	HR: 0.309	(64)
	Tea bread			0.0000739 mg/kg/day	HR: 0.435	(64)
	Sugar bread			0.0000629 mg/kg/day	HR: 0.370	(64)
	Butter bread			0.0000282 mg/kg/day	HR: 0.166	(64)
	Not specified			0.0000525 mg/kg/day	HR: 0.309	(64)
	Tea bread			0.0000739 mg/kg/day	HR: 0.435	(64)
	Sugar bread			0.0000629 mg/kg/day	HR: 0.370	(64)
	Butter bread			0.0000282 mg/kg/day	HR: 0.166	(64)
	Not specified	Heliotrine		0.16 mg/kg	NR	(156)
	Not specified	Heliotrine-N-oxide		5.4 mg/kg	NR	(156)
	Not specified	Lasiocarpine		0.045 mg/kg	NR	(156)
	Not specified	Total pyrrolizidine alkaloids		5.6 mg/kg	NR	(156)
Coffee		Trigonelline		13.8 g/day	NR	(55)
Fermented meat	Salt-fermented pollock	N-nitrosodimethylamine		Range 0.002 to 0.090 g/kg bw/day	CR; 95th percentile: 3.73 × 10^−^5, mean: 1.0 × 10^−^5, av: 1.71 × 10^−6^	(65)
	Chorizo	Thyroid hormones or iodide compounds		Urinary iodine concentrations 530–2,124 mg/l	AOR 1.88	(66)
Various food including fermented products		Benzophenone-type ultraviolet filters: BP, 4-MBP, BP-3, PBZ, 2- OHBP, M2BB, 4-OHBP		20–51 ng/kg/day	NR	(67)
		Furan (adults)		380/494 ng/kg bw/day	MOE > 10,000 for more than 10% of the population and no result < 100	(71)
		Furan (children)		417–421 ng/kg bw/day	MOE: 100–10,000	(70)
		Polychlorinated dibenzo-p-dioxins (PCDDs)1, polychlorinated dibenzofurans (PCDFs) and dioxin-like PCBs (polychlorinated mono-ortho (mo-PCBs), and non-ortho biphenyls (no-PCBs)		0.8 pg WHOTEQ/kg bw/d	NR	(68)
		Sulfite		8.6 to 50 mg/kg	below the maximum permitted level for each food category (max 0.19 mg/kg BW/day)	(69)

NR, not reported; TEQ / WHO-TEQ, Toxic Equivalent / World Health Organization Toxic Equivalent; TDI, Tolerable Daily Intake; bw, body weight; HQ, Hazard Quotient; HR, Hazard Ratio; MOE, Margin of Exposure; CR, Cancer Risk.

In dairy products, including cheese, yogurt, and dairy desserts, several dioxin-like compounds were detected, with estimated average daily intakes ranging from 7.7 to 233 g/day. Although some authors have suggested that fermentation processes may contribute to the degradation of dioxins, Loutfy et al. (61) observed that, in the case of cheese, concentrations of dioxins and dioxin-like contaminants exceeded the acceptable limits. Risk characterization indicated a potential health concern, as exposures remained above the tolerable daily intake (TDI) established for these compounds. Similarly, bisphenol A (BPA) was detected in cheese and yogurt at concentrations ranging from 521 to 640 ng/mL. Although the calculated hazard quotient (HQ) suggested a need for consideration of non-carcinogenic risks. Among other hazardous organic compounds detected in dairy products are antimicrobial residues. In the study by Alenezi et al. (62), residues of ampicillin, tetracycline, oxytetracycline, and amoxicillin were found at concentrations ranging from 2.44 to 220.3 μg/L. However, the overall risk associated with these antibiotic residues in dairy products was considered low.

In bread and bakery products, potassium bromate (KBrO3) was frequently identified as the main contaminant. Concentrations ranged from 0.0000282 to 49.19 mg/kg/day depending on the product type, with the highest values observed in wheat bread. The corresponding hazard ratios (HRs) varied widely, ranging from 0.166 to over 295,000. A significantly higher risk was observed for consumption of bread from Cameroon (63) than for the studied products from Ghana (64).

In fermented meat products, notable contaminants included N-nitrosodimethylamine (NDMA) in salt-fermented pollock, with estimated exposures of 0.002–0.090 g/kg bw/day. Monte Carlo-based cancer risk simulations (mean = 1.0 × 10^−^5; 95th percentile = 3.73 × 10^−^5) indicated a low but measurable carcinogenic potential associated with frequent consumption (65). Additionally, elevated urinary iodine concentrations (530–2,124 mg/L) were observed in consumers of chorizo, suggesting a possible association with thyroid hormone dysregulation due to iodide or thyroid-active compounds (AOR = 1.88) (66).

In several studies (67–71), different types of foods, including fermented products, were analyzed collectively, rather than as separate categories. Consequently, the detected contaminants reflected the combined diversity of the sampled products rather than the specific composition of fermented foods. Reported substances included benzophenone-type ultraviolet filters (BP, BP-3, PBZ, 4-MBP), furan, and pyrrolizidine alkaloids, all occurring at levels generally below toxicological concern for adults. However, for children, some MOE values for furan exposure approached the 100–10,000 range, suggesting a potential concern for sensitive groups (70).

Coffee was mentioned in a single study as a source of trigonelline, a natural alkaloid with an estimated daily intake of approximately 13.8 g/day, which was not associated with any toxic or adverse health effects (55).

Two additional studies included products that could potentially be categorized as fermented, although this could not be confirmed based on the available descriptions. The study by Han et al. (72) investigated homemade sausages and reported the presence of sodium nitrite, with a very low MOE. The reported dose of 3.5 g/kg was extremely high compared with intake standards (maximum 125 mg/kg in the final product). Similarly, in the study of Cvetković et al. (73) on millet-based products, atropine and scopolamine were detected at concentrations of 0.23 ng/kg bw/day.

For several other studies no quantitative data on intake or risk characterization were provided. Nevertheless, the analyzed fermented products can be considered in the context of their possible contamination with various compounds, such as verotoxin in yogurt (74), caffeine in coffee (75), or phthalates in bread (75).

## 4 Discussion

This scoping systematic analysis was designed to answer two complementary questions: “Are fermented foods safe for humans?” and “What are the main microbiological and chemical hazards posed by fermented foods and the associated risks?”.

Fermentation is widely recognized as a food-processing strategy that can enhance safety and stability through technological approaches such as acidification, salt addition, reduced water activity, production of antimicrobial metabolites, and microbial competition. These properties can inhibit or suppress many pathogenic and spoilage organisms, contributing to safer foods and extended shelf-life (76). Fermented foods are considered an established diet component with a generally favorable safety profile, despite safety depending on ingredient quality and manufacturing conditions rather than on fermentation alone (77), as empirically evidenced by the collected data: the microbiological hazards and outbreaks identified were rarely interpretable as being caused by fermentation itself; instead, they aligned with entry via the substrate (e.g., contaminated milk, meat, cereals, vegetables, fish) or with manufacturing practices and post-processing contamination. This perspective is particularly important for food products for which there is no final step to decrease the associated risk (e.g., many cheeses and cured meats), as the microbiological quality of the production environment and raw materials becomes a decisive factor in determining risk.

Across the retrieved literature, bacteria were the most frequently reported microbiological hazards, with recurrent reporting of *Salmonella* spp., pathogenic *Escherichia coli* and other *Enterobacteriaceae, Campylobacter jejuni*, and *Clostridium botulinum*. *Listeria monocytogenes* appeared less frequently but was associated with the most severe outcomes, consistent with its high case fatality in vulnerable groups. The product group most often implicated was fermented dairy, especially cheeses and yogurt, followed by selected fermented meats, with sporadic entries in vegetable, cereal-based, fish, and mixed dishes containing fermented ingredients. Despite considerable heterogeneity in the context, a pattern was identified: hazards tend to enter early (raw milk or insufficiently controlled ingredients), persist when fermentation parameters are insufficient to suppress them, or be introduced after fermentation (e.g., slicing/handling, mixed dishes). This pattern is also consistent with the role of starter cultures as a safety tool, considering that well-selected cultures can reduce opportunities for pathogen growth, but they cannot compensate for heavily contaminated substrates or poor hygienic conditions (77). A key implication is the heightened relevance of production contexts. This is especially true in traditional or household fermentations. In these contexts, sanitation and temperature control are more variable (78). In such contexts, safety margins can narrow substantially, increasing the likelihood that pathogens or toxin-producing organisms present in the substrate will survive or proliferate. However, insufficient evidence was found to establish a clear picture of the relationship between production contexts and the associated risk of fermented foods. This is consistent with broader evidence on minimally processed foods, including plant-based foods, where inadequate hygienic practices during preparation and handling have similarly been shown to represent a primary driver of bacteriological contamination and associated public health risk (79).

Across the studies retrieved in our systematic analysis, chemical hazards were also reported across diverse fermented foods, namely mycotoxins, especially in cereal-based foods and dairy products, heavy metals, with product- and geography-specific drivers, process-related contaminants, e.g., PAHs and acrylamide in baked/smoked matrices, and a smaller set of other compounds, such as furan, UV filters and nitrosamines in specific contexts. The chemical hazards reported in fermented foods can be classified within two broad, policy-relevant categories: (i) hazards primarily driven by the food matrix and upstream contamination and (ii) hazards that may be influenced by processing choices, including fermentation-related pathways. This distinction is critical for understanding whether fermented foods are “safe”: the data indicate that most chemical hazards are not inherently created by fermentation but rather reflect contamination present in raw materials and primary production environments, with fermentation sometimes modifying, rather than initiating, exposure. In fact, fermentation and downstream processing can change concentrations by degradation, binding, redistribution, or concentration via moisture loss, so a matrix-based hazard can still be relevant at the consumption level (80).

Mycotoxins represent one of the clearest examples of hazards primarily driven by upstream contamination rather than fermentation biology. In the broader literature, mycotoxins in fermented products are largely attributable to fungal contamination of ingredients before processing, including during storage, and can persist because many mycotoxins are relatively heat-stable and may survive standard processing conditions (13). This framing aligns with the pattern observed in the obtained results, in which mycotoxin-related findings were concentrated in cereal-based products (where grain quality and storage are decisive) and in dairy, where aflatoxin M1 reflects carry-over from contaminated animal feed into milk and derived products (12). It is also important to mention that the fermentation can sometimes reduce mycotoxin concentrations through microbial binding or biotransformation, but these effects are variable and depend on strain, matrix, and process parameters, and, therefore, fermentation should not be assumed to be uniformly protective (13). From a risk management perspective, it is clear that the most effective strategy is to ensure the safety of raw materials (through good agricultural practices, storage control, supplier verification and targeted monitoring), rather than relying on fermentation to remediate contaminated ingredients (81). This supports that many chemical hazards associated with fermented foods reflect the food system upstream rather than an inherent hazard generated by fermentation.

The literature indicates that metal concentrations in dairy and other fermented matrices are primarily determined by upstream and processing-chain factors, particularly environmental contamination of raw materials and potential contributions from contact materials, rather than by microbial fermentation processes (53, 82). Metals emerged in the obtained results as a highly context-dependent hazard class, with risk characterization varying substantially by product type, geographic origin, and population subgroup (e.g., differences driven by consumption patterns and body weight assumptions in children and adults). This variability is consistent with broader evidence indicating that metal occurrence in milk and dairy products is shaped primarily by upstream environmental determinants, including contamination of soil, water, and feed, together with potential concentration effects during processing and differences in local production systems, thereby motivating structured monitoring and risk assessment across supply chains (53, 82).

For the substances considered process-derived contaminants, the fermentation of the food is secondary to the contribution of high-temperature processing and/or smoking within the overall manufacturing chain. PAHs exemplify this distinction since they arise primarily from incomplete combustion and smoke deposition, and their occurrence in foods is therefore governed mainly by smoking-related variables (e.g., wood type, smoke-generation temperature and airflow, smoking duration, distance to the heat source, fat content and casing, and equipment design and maintenance), rather than by microbial fermentation pathways (83, 84). Thus, PAHs reported in fermented products (e.g., smoked fermented meat products) should be interpreted as reflecting the smoking unit operation and its exposure conditions, not as hazards generated by fermentation. This is consistent with the previous published evidence showing that PAH profiles and loads in thermally processed meat products are largely driven by processing parameters and smoking technology (85).

Acrylamide provides a parallel example of a contaminant determined primarily by thermal processing rather than fermentation. Acrylamide formation is dominated by Maillard chemistry during baking or roasting, shaped by precursor availability (particularly free asparagine and reducing sugars), temperature and time profiles and moisture conditions. Accordingly, it is best conceptualized as a thermal-processing hazard (86). Nonetheless, fermentation can modulate acrylamide formation indirectly, most notably in sourdough systems, by altering precursor pools and dough pH, thus serving as a potential mitigation strategy within an otherwise heat-driven pathway (86, 87). Thus, the evidence indicates that when PAHs and acrylamide are reported in products classified as fermented, their occurrence should be interpreted primarily as a function of co-occurring unit operations in the manufacturing chain (e.g., smoking, baking, roasting) and their associated process conditions, rather than being attributed to microbial fermentation as the causal determinant.

Another example is Furan, a recognized heat-induced processing contaminant, reported especially in coffee and heat-treated foods. While coffee may be consumed in fermented-product contexts (e.g., fermented coffee processing), furan formation is primarily driven by roasting and thermal degradation of precursors rather than fermentation (88). As reflected in the obtained results, this again supports the broader conclusion that several hazards associated with fermented foods are, in practice, indicators of thermal processing and product-specific manufacturing chains. Nitrosamines are relevant to specific product categories, most notably cured meats, where nitrite and/or nitrate use, processing, and storage conditions influence formation pathways. Regulatory and scientific bodies have highlighted nitrosamines as genotoxic carcinogens of concern in foods, supporting the need for continued mitigation and surveillance in relevant processed meat chains (89). Recent literature has also summarized formation mechanisms and control strategies, framing nitrosamines as a hazard shaped by formulation and processing conditions rather than by fermentation as a microbial transformation alone (90).

A small number of studies addressed compounds such as UV filters in specific contexts. These are best interpreted as emerging contaminants potentially linked to packaging materials, environmental contamination, or complex supply-chain exposures rather than fermentation itself. Evidence highlights that migration and contamination depend on packaging composition, food properties (fat content, acidity), time-temperature, and regulatory compliance, underscoring the importance of considering packaging as part of the overall risk system (90, 91). Given the emerging nature of these hazards and the fragmented reporting landscape, a lack of retrieved findings should not be interpreted as the absence of risk, but rather as potentially reflecting limited data availability and under-investigation at present.

Among the chemical hazards identified, biogenic amines (BAs) represent the clearest example of a hazard that can be linked directly to the fermentation ecosystem, namely, the presence and predominance of decarboxylase-positive microorganisms, the availability of free amino acid precursors, and process conditions that permit microbial growth and enzymatic activity (92, 93). BAs such as histamine, tyramine, putrescine and cadaverine are formed through microbial amino acid decarboxylation, and their accumulation is therefore highly matrix-dependent, being most relevant in protein-rich foods (e.g., cheeses, fermented meats, fish sauces) where precursor amino acids are abundant and where ripening and storage can provide time for amine production (11, 93). Importantly, the BA hazard is not an inherent or inevitable consequence of fermentation, but rather a function of controllable ecological and technological factors. Hygienic quality of the raw materials and processing environment (which influences the initial load and diversity of BA-producing microorganisms), the choice of starter cultures (including avoidance of decarboxylase-positive strains and, where appropriate, use of strains with amine-degrading capacity), and the management of critical parameters such as temperature, salt concentration, pH and acidification kinetics and oxygen availability, which collectively determine whether BA-formers can proliferate and express decarboxylase activity (92, 93). Accordingly, BA formation is best framed as a predictable and manageable chemical risk within fermented foods, for which risk reduction can be achieved through validated processing design and verification, reinforcing that hazards attributed to fermented foods frequently reflect upstream or process-control determinants rather than fermentation as an intrinsically hazardous step (11, 92).

Taken together, the evidence gathered in the present systematic review supports a coherent and practically useful interpretation of safety in fermented foods. The hazard landscape is largely structured by where hazards enter the chain and which unit operations dominate exposure, rather than by fermentation as an intrinsically hazardous technology. Across food categories, the most frequently reported chemical risks are consistent with i) upstream contaminants that originate in primary production and raw materials (e.g., mycotoxins and metals), and ii) process-derived contaminants associated with smoking or high-temperature steps that may co-occur with fermentation (e.g., PAHs, acrylamide, furan). In contrast, the subset of hazards most plausibly linked to fermentation is comparatively narrow, with BAs representing the most emblematic example, driven by the presence of decarboxylase-positive microorganisms, precursor availability and permissive processing conditions. This aligns with recent evidence emphasizing both the long history of safe consumption and the need to interpret hazards through a product- and process-specific lens, rather than attributing risk generically to fermentation (1, 12). Additionally, production context is a major determinant of both microbiological and chemical hazard profiles, yet it is insufficiently captured in the published literature in a way that supports structured comparison. The current evidence is most often reported by food category, while information on whether the product was produced in a household, artisanal, or industrial setting is either absent, inconsistently described, or not operationalised in risk characterization. This matters because the same fermented food category can embody markedly different safety envelopes depending on context. Household fermentation may involve higher variability in hygiene, temperature control, salt application, and fermentation kinetics, artisanal production can be highly heterogeneous, sometimes combining traditional practices with variable degrees of standardization and industrial processes typically rely on defined starters, validated time, temperature, salt and pH targets, and environmental monitoring schemes. These contextual differences plausibly influence not only pathogen entry and persistence (i.e., through raw-material handling, sanitation, cross-contamination, and post-process exposure), but also the formation and accumulation of certain fermentation-linked metabolites such as BAs, which are strongly shaped by microbial ecology and process conditions (10, 11, 93). Consequently, future research would benefit from moving beyond categorization by food type alone and adopting a standardized reporting framework that systematically captures production setting, critical control parameters, starter culture use (including strain-level characterization where relevant), and post-fermentation handling conditions. Such harmonization would improve comparability across studies and enable a more rigorous differentiation between hazards attributable to contaminated inputs, process deviations, and fermentation ecology. The same approach would also strengthen the interpretability of risk metrics by anchoring them to transparent intake scenarios and population subgroups and by clarifying whether reported values reflect typical manufacturing practices or exceptional conditions. This is particularly relevant for hazards that are demonstrably upstream-driven, such as metals and mycotoxins, where supply-chain context and environmental determinants dominate concentrations in the finished product, and where relying on downstream processing to offset contaminated inputs is unlikely to be a robust safety strategy. Finally, the increasing uptake of genomic and high-resolution analytical methods offers a timely opportunity to improve attribution and verification in complex fermentation environments, supporting more precise identification of hazards, routes of contamination, and the effectiveness of controls (94).

Overall, these considerations reinforce the rationale that the available evidence, accumulated over years of surveillance and research, consistently points to contaminated raw materials, inadequate process control, and post-fermentation handling as the primary drivers of risk in fermented foods, rather than fermentation as an intrinsically hazardous technology. This provides a robust and coherent characterization of the hazard landscape of this food category.

The increasing diversity of novel fermented foods represents an emerging challenge that deserves explicit attention in future food safety research. Fermented plant-based foods, alternative protein sources, including legume- and cereal-based fermented matrices, and products derived from precision fermentation or produced with novel starter cultures are gaining considerable market relevance, yet their safety profiles remain incompletely characterized (15). These products may introduce new combinations of substrates, microorganisms and processing conditions for which the existing evidence base is limited or absent (15, 95). The use of novel or non-conventional starter cultures raises questions about the potential for unexpected metabolite formation, including biogenic amines or other fermentation-derived compounds, under conditions that have not yet been systematically studied (96). Food safety frameworks and surveillance systems will need to adapt proactively to capture these emerging risks, and future systematic reviews in this area should explicitly include novel fermented food categories as their evidence base matures.

## 5 Study limitations and sources of uncertainty

Several limitations should be considered when interpreting the findings of this review. The included studies are heterogeneous in design, spanning outbreak investigations, case reports, observational studies, and quantitative exposure assessments, and no formal quality appraisal was applied, as the diversity of designs precluded the use of a single standardized tool. Thus, it should be recognized that not all included studies contribute equally to the strength of the conclusions drawn. Publication bias represents an additional source of uncertainty. The capacity to detect, investigate, and publish outbreak data varies considerably across countries and surveillance systems, and the absence of reported outbreaks for a given food category should not be interpreted as direct evidence of safety. The quantitative risk estimates reported, particularly for chemical hazards, stem from studies that use different populations, dietary surveys, analytical methods, and exposure models, making direct cross-study comparisons difficult. Finally, the evidence base is predominantly derived from established food categories and may not fully capture the safety profile of traditional, household, or emerging fermented food products.

## 6 Conclusions

These results illustrate the complex balance required to assess dietary exposure to heavy metals in fermented foods. While levels of some metals approach the thresholds of concern, the overall risk indices, supported by biomonitoring evidence, indicate risks that can be managed with sensible dietary choices. This analysis highlights the importance of continued monitoring, refining risk assessments, and considering vulnerable populations in food safety and regulatory frameworks.
